# Milk fat globule size development in the mammary epithelial cell: a potential role for ether phosphatidylethanolamine

**DOI:** 10.1038/s41598-020-69036-5

**Published:** 2020-07-23

**Authors:** Leonie Walter, Vinod K. Narayana, Richard Fry, Amy Logan, Dedreia Tull, Brian Leury

**Affiliations:** 1 Faculty of Veterinary and Agricultural Sciences, The University of Melbourne, Building 184 Royal Parade, Parkville, VIC 3010 Australia; 2 CSIRO Agriculture and Food, 671 Sneydes Rd, Werribee, VIC 3030 Australia; 3 Metabolomics Australia, Bio21 Institute of Molecular Science and Biotechnology, The University of Melbourne, Parkville, VIC 3052 Australia

**Keywords:** Lipidomics, Membrane lipids, Animal physiology

## Abstract

Milk fat globule (MFG) size is a milk production trait characteristic to the individual animal and has important effects on the functional and nutritional properties of milk. Although the regulation of MFG size in the mammary epithelial cell is not fully understood, lipid droplet (LD) fusion prior to secretion is believed to play a role. We selected cows that consistently produced milk with predominantly small or large MFGs to compare their lipidomic profiles, with focus on the polar lipid fraction. The polar lipid composition of the monolayer surrounding the LD is believed to either promote or prevent LD fusion. Using a targeted LC–MS/MS approach we studied the relative abundance of 301 detected species and found significant differences between the studied groups. Here we show that the lipidomic profile of milk from small MFG cows is characterised by higher phosphatidylcholine to phosphatidylethanolamine ratios. In contrast, the milk from large MFG cows contained more ether-phosphatidylethanolamine species. This is the first time that a potential role for ether-phosphatidylethanolamine in MFG size development has been suggested.

## Introduction

Milk lipids are secreted from the mammary epithelial cell as spherical structures termed milk fat globules (MFGs). They comprise a neutral lipid core surrounded by a membrane derived partially from the endoplasmic reticulum (ER, innermost layer) and the apical plasma membrane (two outer layers)^1^. The MFG membrane (MFGM) keeps lipids solubilised in milk, but also has several health benefits for infant and adult consumers. For example, beneficial effects on cancer, hypercholesterolemia, diabetes and cognitive function have been attributed to the protein, glycerophospholipid (PL) and sphingolipid (SL) components of the MFGM in bovine milk^2^. The amount of MFGM material in milk depends on the fat content and MFG size, with the greater surface area in smaller globules providing more MFGM material per unit of fat^3^. Moreover, the processing of milk into butter and cheese is influenced by the MFG size. For example, larger MFGs are more prone to coalescence and can increase the efficiency of butter manufacturing, while cheese manufactured from milk with small MFGs can have improved sensory properties^4^.

**Figure 1:**
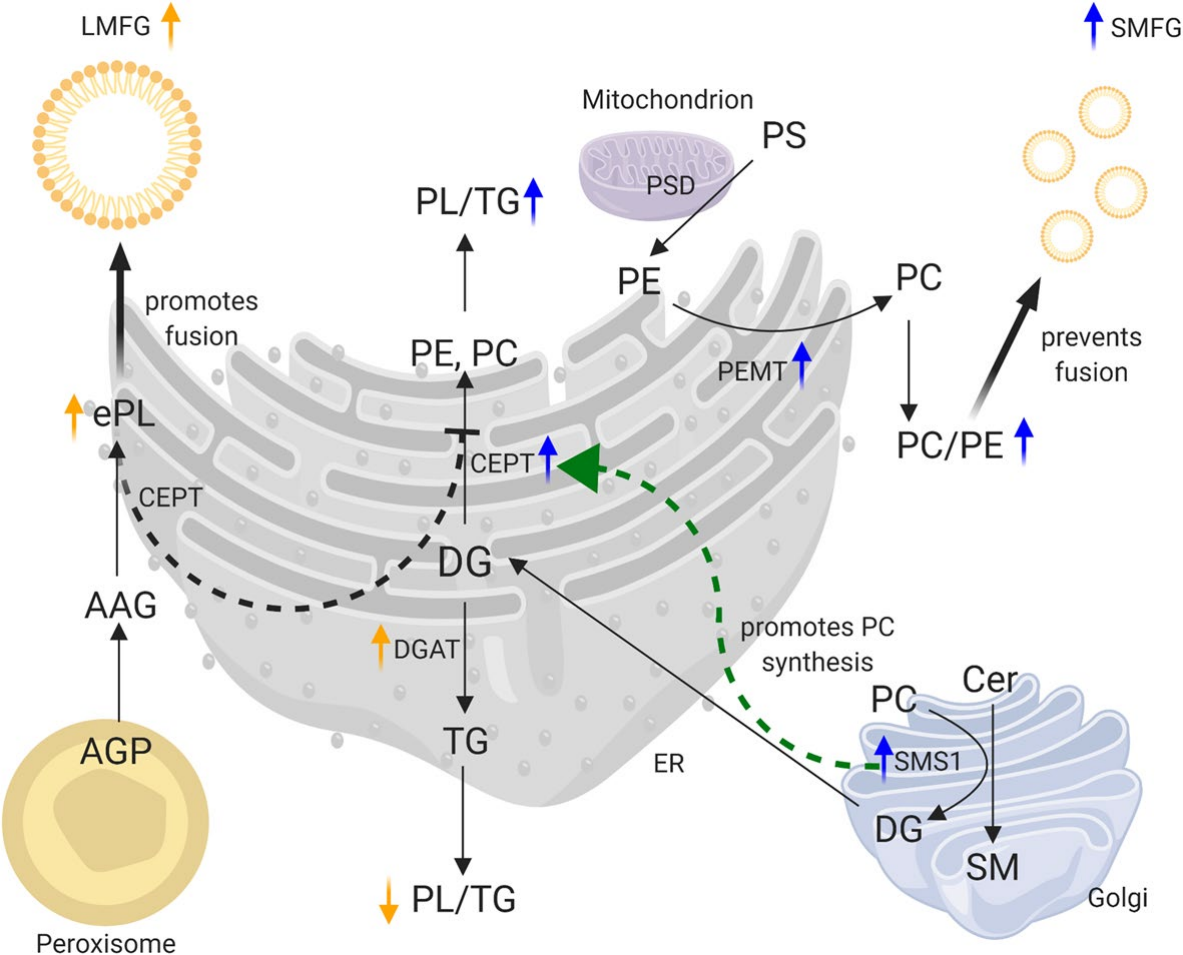
Possible mechanisms through which major metabolic pathways contribute to the milk fat globule (MFG) size development in the mammary epithelial cell. This illustration is based on the currently available literature and provides hypothetical explanations for the observed differences in milk lipidomic profiles between the studied groups. Please note that the precise role of these metabolic pathways has not been established. Diacylglycerol (DG) plays a central role and connects phospholipid (PL), triacylglycerol (TG) and sphingolipid synthesis pathways. DG is utilised to either produce TG or the major PLs, phosphatidylethanolamine (PE) and phosphatidylcholine (PC). TG synthesis is catalysed by the enzyme diacylglycerol-acyltransferase (DGAT), in the form of two isoenzymes DGAT1 and DGAT2 which are located in the endoplasmic reticulum (ER) or on the surface of lipid droplets (LD), respectively^5^. The synthesis of PC and PE is catalysed by DG choline/ethanolamine phosphotransferase (CEPT) found in the ER^61^. This enzyme also catalyses the production of ether PLs (ePL) by transferring a choline or ethanolamine headgroup to alkylacylglycerol (AAG), which is derived from peroxisomal production of 1-O-alkyl-glycerol-3-phosphate (AGP)^33^. We suggest that when DG is more efficiently channelled towards TG synthesis, for example due to increased DGAT1 activity, diacyl-PL synthesis could be limited and ePL synthesis through CEPT in the ER could present a possible salvage pathway that is not reliant on DG as a precursor. This shift towards ePL synthesis could promote LD fusion and thus contribute to the large MFG (LMFG) phenotype. In the small MFG (SMFG) phenotype, DG could be more efficiently channelled towards PL synthesis, either due to decreased DGAT1 activity or possibly due to increased SM synthase 1 (SMS1) activity in the Golgi apparatus, although this hypothesis could not be confirmed by the current study. SMS1 catalyses the transferal of the choline headgroup from PC to ceramide (Cer) to produce SM and DG, thus contributing to the DG pool. Moreover, increased SMS1 activity can stimulate PC synthesis^51^. Another potential pathway contributing to the SMFG phenotype is the phosphatidylethanolamine *N*-methyltransferase (PEMT) pathway, which converts PE into PC and thus increases the PC/PE ratio. The PE used in the PEMT pathway is derived mainly through decarboxylation of phosphatidylserine (PS) in the mitochondrion by PS decarboxylase (PSD) and results in PC with a higher degree of unsaturation. Increased unsaturation within the monolayer surrounding the LD and increased PC/PE ratios could prevent LD fusion, resulting in the SMFG phenotype. Figure created with BioRender.com.

The formation of MFGs within the mammary epithelial cell starts with the accumulation of neutral lipids (mainly triacylglycerols (TG)) in the ER^1^. This results in the formation of a lipid droplet (LD), the intracellular precursor of the MFG, which is released from the ER surrounded by a single layer of polar lipids and membrane proteins. The ultimate size of individual MFGs is determined by two growth mechanisms inside the mammary epithelial cell: either via local TG synthesis by enzymes residing in the monolayer surrounding the LD or by LD fusion^5,6^. The exact mechanisms behind LD fusion remain elusive but may depend on the PL and fatty acid (FA) composition of the monolayer. PLs with smaller headgroups, such as phosphatidylethanolamine (PE) and phosphatidic acid are predicted to enhance fusion of LD, while phosphatidylcholine (PC), which has a more cylindrical shape, can protect LDs from fusion^7^. Although most of the available evidence in the literature is based on studies in non-mammary cells, this important role of the polar lipid composition of the LD monolayer in the regulation of LD fusion has also been demonstrated in mammary epithelial cells^8^. Moreover, not only the type but also the FA composition of the polar lipids residing in the monolayer surrounding the LD determine its size^9^. Apart from sphingomyelin (SM), which contains predominantly saturated FAs (SFAs), most PLs are rich in unsaturated FAs (UFAs), although PC is less unsaturated compared to PE, phosphatidylinositol (PI) and phosphatidylserine (PS)^10^. Using a cell line engineered to produce larger LDs, it has been shown that LDs extracted from these cells contained less UFAs in the PE and PC fractions of the LD monolayer compared to control cells with smaller LDs^9^. Figure 1 illustrates the suggested impact of several metabolic pathways on LD and ultimately MFG size, based on evidence from the literature combined with hypothetical explanations for the differences in lipidomic profiles reported in the current study.

Variations in MFG size exist between species of mammals and between breeds of the same species^11,12^, but also between animals of the same breed of cows^13,14^. The size of MFGs in cow’s milk ranges from less than 1 µm to 15 µm^15^, with the average size ranging between 2.5 and 5.7 µm within a herd of Holstein–Friesian cows^13^. Moreover, the average MFG size changes throughout the lactation cycle depending on the days in milk since the last calving and throughout the year in response to changes in the diet^16^. Other suggested impact factors on MFG size are fat content^17^, fat-to-protein ratio^18^ and milking period^16^. MFG size also depends on the physiological state of the animal^16,19^ and the milk from cows producing small or large MFGs can differ in the FA composition^14,18^. However, the magnitude of the effects of parameters such as the physiological state of the animal and its milk production traits on MFG size is less than the overall variation that is seen between individual animals^20^.

Differences in the FA composition of MFGs from cows naturally producing milk with small or large average MFG sizes have previously been reported^14^ and were also demonstrated in our previous study using the same samples as in the current work^21^. Moreover, a role of the relative proportion of major polar lipid classes in milk fat, such as PE and PC, is believed to impact MFG size^22–24^, although this has only been shown using milk samples separated into small and large MFG fractions and is yet to be demonstrated using whole milk samples from cows that naturally produce milk with small or large average MFG sizes. We hypothesised that lipidomic differences between cows consistently producing small or large MFGs could extend beyond the major lipid classes and therefore aimed to study the role of minor lipid classes and individual lipid species on MFG size using triple quadrupole LC–MS/MS analysis. To the best of our knowledge, with 301 detected species, this study presents the most extensive milk lipidomic analysis yet and reports differences in the polar lipid species and their FA composition between cows producing small or large MFGs.

## Results

### Differences in milk production traits and MFG size between the studied groups

The average MFG size, expressed as the volume weighted mean diameter D_4,3_, was 1.63 µm larger in the milk from cows in the large MFG (LMFG) group compared to the small MFG (SMFG) group. The selected cows also differed significantly in the surface weighted mean diameter D_3,2_ (Table 1). Other production traits such as milk composition, the average number of milking sessions per day and concentrate intake were statistically similar between the studied groups (Table 1). However, there was a small difference in days in milk, representing the number of days since the last calving (25 days difference, Table 1). Furthermore, cows from the SMFG group included some primiparous cows (parity 1 to 3), while the LMFG group contained cows in later parities only (parity 3 to 5).

**Table 1:** Milk fat globule (MFG) size, milk composition and animal production data of the selected cows producing milk with small (SMFG) or large (LMFG) MFG size distributions, expressed as volume-weighted mean diameters (D_4,3_) and surface-weighted mean diameters (D_3,2_).

	SMFG Cow		LMFG Cow		*P* value
	Mean	95% confidence interval	Mean	95% confidence interval	
MFG size D_4,3_ (µm)	3.29	2.96, 3.62	4.92	4.58, 5.25	0.003
MFG size D_3,2_ (µm)	2.80	2.51, 3.09	4.04	3.53, 4.55	0.003
Days in milk	168.3	117.0, 219.7	143.3	92.0, 194.7	0.164
Parity	1.8	1.1, 2.6	3.8	3.1, 4.6	0.028
Concentrate intake (kg/day)	8.6	6.2, 11.1	8.4	5.9, 10.9	0.775
Milkings (sessions/day)	2.8	2.4, 3.2	2.8	2.4, 3.2	0.854
Milk yield (kg/day)	27.84	19.41, 36.27	29.25	20.82, 37.68	0.854
Fat content (%)	3.40	2.56, 4.25	3.77	2.93, 4.62	0.729
Fat yield (kg/day)	0.92	0.76, 1.07	1.01	0.86, 1.16	0.729
Protein content (%)	2.96	2.76, 3.16	3.00	2.81, 3.20	0.729
Fat/protein ratio	1.15	0.92, 1.37	1.24	1.01, 1.47	0.729

Statistical significance was determined by two-way ANOVA including the studied groups (LMFG and SMFG cows), days in milk and season as fixed effects, except for DIM where only group and season were included as fixed effects. *P* values were adjusted for multiple comparisons using the Benjamini and Hochberg^58^ procedure. All parameters represent the respective unit in the previous 24 h and were recorded after the milking during which the samples were taken. Exceptions are days in milk and parity, which represent the day of sampling and milkings which represents the average milking visits per cow per day over the previous week. The data presented in this table is also presented as part of another study using milk samples from the same cows selected for the current study which were collected at the same time^21^.

### Lipidomic profile of milk from the studied groups

Lipidomic analysis was performed on whole raw milk samples which were frozen within a minute of sample collection and thus most accurately represent the milk lipid metabolite composition of each animal at the time of sampling. Using a targeted approach, we measured 445 compounds and detected 301 thereof in milk. Although previous reports have reported higher numbers of neutral lipid species^25^, to the best of our knowledge, this study presents the highest number of polar lipids detected in milk to date. These species were from a total of 14 lipid classes, many of which had not been previously the focus of milk lipidomics (Table 2). For example, we detected 23 and 19 species of plasmenyl-PE and plasmenyl-PC, respectively. Moreover, we detected 24 ceramide (Cer) species and five and seven additional dihydroceramide and hexosylceramide species. Detected lysophospholipid species included 18 lysophosphatidylcholine (LPC), six lysophosphatidylethanolamine (LPE) and three lysophosphatidylinositol (LPI) species (Table 2). A complete list of the identified lipid species is provided in the supplementary file of this article. Amongst the detected compounds, nine lipid species were significantly different in relative abundance (*P* < 0.05) and 18 species tended to differ (*P* < 0.10) between the SMFG and LMFG groups.

**Table 2:** Lipid classes surveyed and detected in the current study.

Lipid class	Number of species surveyed	Number of species detected
PC	33	25
PC-O	19	15
PC-P	18	14
PE	19	17
PE-O	10	9
PE-P	33	23
PI	16	15
PS	7	5
PG	4	3
SM	35	33
CE	27	6
oxCE	2	1
DG	20	18
TG	44	44
TG-O	3	3
Cer	42	24
dhCer	6	5
HexCer	30	7
LPC	37	14
LPC-O	10	3
LPC-P	4	1
LPE	10	5
LPE-O	-	-
LPE-P	4	1
LPI	4	3
AC	12	4
Sulfatide	6	1
Ganglioside	8	1

dhCer, dihydroceramide; HexCer, Hexosylceramide; AC, acyl carnitine; oxCE, oxidised CE; PG: phosphatidylglycerol; TG-O, can include alkyl-diacylglycerol, dialkyl-acylglycerol or trialkylglycerol.

To date, about 400 lipid species have been discovered in milk, however the estimated total number is believed to be around 1,000 lipid species, with many low abundance compounds yet to be discovered^26^. The targeted method used in our study allowed us to identify the total carbon number (CN), number of double bonds and the FA composition of glycerophospholipids which was characterised by collision induced dissociation in positive ionization mode as reported previously^27,28^. However, cow’s milk contains short chain FAs with less than 14 carbons^29^ and TG species containing these FAs, resulting in a total CN < 48, which will not be detected by this approach. This represents a limitation of the present study in relation to TG analysis since a large proportion of TGs have total CNs < 48:1 which was the smallest CN of TG species detected in our study^26^. Therefore, the differences in the FA composition and origin of FAs between the studied groups were not the focus of the current study. The PL components of milk, on the other hand, contain mostly medium and long chain FAs and the lipidomic approach described here covers the major lipid species in this group. Therefore, the focus of this study was the identification of differences in polar lipid species.

### Differences in neutral lipid species

**Figure 2:**
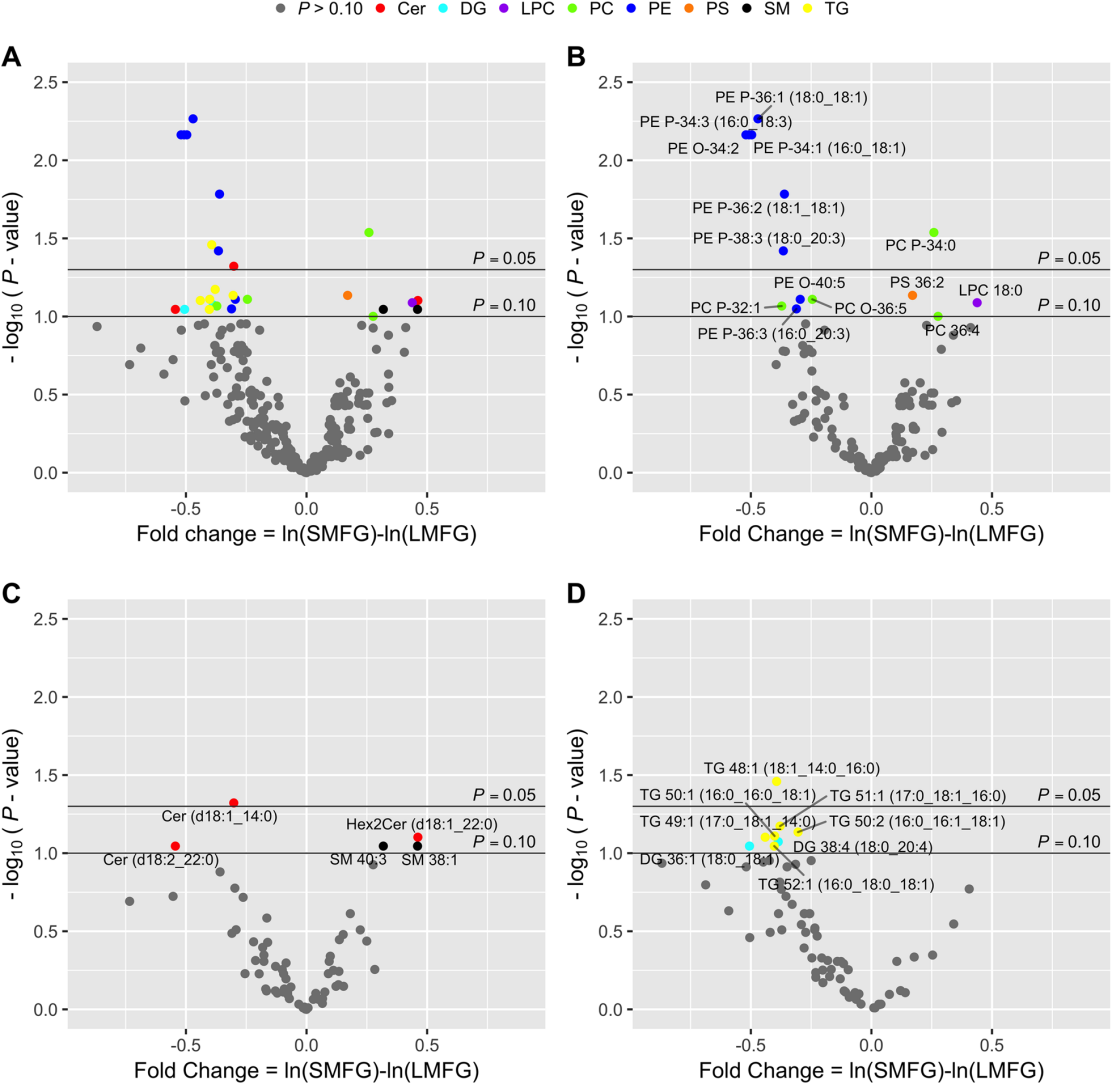
Volcano plots showing the milk lipidome of cows with small (SMFG) or large (LMFG) average milk fat globule size. The abundance of lipid species from SMFG relative to LMFG milk are shown as (**A**) total lipid species, (**B**) phospholipid species, (**C**) sphingolipid species and (**D**) neutral lipid species with a total carbon number of 48 and over. The fold change, expressed as ln(SMFG)-ln(LMFG), when multiplied by 100 represents the change in %. Lipid species with positive percentages are more abundant in SMFG milk and lipid species with negative percentages are more abundant in LMFG milk. The horizontal lines denote statistical significance with lipid species above the upper line displaying adjusted *P* values < 0.05 and above the lower line displaying adjusted *P* values < 0.10. *P *values were adjusted according to the Benjamini and Hochberg^58^ procedure.

The milk lipidomic profile contained six TG lipid species that were relatively more abundant in LMFG milk compared to SMFG milk (Fig. 2D). However, detailed analysis of the lipid species revealed that the TGs with higher relative abundance in whole milk of LMFG cows mainly contained FAs with one or no double bond (*P* = 0.132, Table 3). Although, the TG classes measured in this study were limited to species with CN ≥ 48, these results align with the results reported by others^14^. Therefore, the results of the milk lipidome in combinations with previously reported results^14,18^, indicate higher saturation in LMFG cows compared to SMFG cows, while fat yield and fat content were statistically similar between the groups (Table 1).

**Table 3:** Relative abundance of lipid species with different degrees of saturation in the milk lipidome of cows producing milk with small (SMFG) or large (LMFG) milk fat globules.

Lipid class	SFA			1 DB			≥ 2 DB		
	Fold change	95% confidence interval	*P* value	Fold change	95% confidence interval	*P* value	Fold change	95% Confidence interval	*P* value
PC	0.10	− 0.09, 0.30	0.533	0.07	− 0.08, 0.22	0.554	0.24	0.04, 0.43	0.132
PE	0.17	− 0.17, 0.51	0.550	− 0.07	− 0.23, 0.10	0.585	− 0.04	− 0.16, 0.08	0.592
PI	− 0.29	− 0.71, 0.13	0.353	0.02	− 0.26, 0.30	0.903	0.15	0.03, 0.27	0.132
PS	–	–	–	− 0.01	− 0.17, 0.14	0.903	0.06	− 0.02, 0.15	0.353
PG	–	–	–	− 0.06	− 0.43, 0.31	0.823	0.15	− 0.10, 0.39	0.467
SM	0.02	− 0.16, 0.20	0.866	0.07	− 0.12, 0.25	0.585	0.13	− 0.03, 0.28	0.330
Cer	− 0.27	− 0.54, 0.00	0.230	0.01	− 0.26, 0.28	0.946	− 0.17	− 0.51, 0.17	0.549
LPC	0.22	− 0.33, 0.77	0.585	0.18	− 0.07, 0.44	0.353	0.34	0.10, 0.58	0.132
LPE	0.03	− 0.35, 0.40	0.903	0.11	− 0.38, 0.61	0.727	0.30	− 0.43, 1.02	0.585
LPI	− 0.37	− 0.68, − 0.06	0.132	− 0.17	− 0.53, 0.19	0.554	0.07	− 0.19, 0.33	0.668
AC	− 0.16	− 0.62, 0.31	0.592	–	–	–	–	–	–
CE	− 0.36	− 0.83, 0.11	0.353	− 0.47	− 1.15, 0.21	0.353	− 0.57	− 0.95, − 0.19	0.132
DG	− 0.14	− 0.50, 0.23	0.585	− 0.25	− 0.55, 0.05	0.330	0.11	− 0.22, 0.44	0.592
TG	− 0.37	− 0.67, − 0.07	0.132	− 0.40	− 0.63, 0.16	0.132	− 0.22	− 0.48, 0.04	0.330

The data is categorised based on the total number of double bonds (DB) in the lipid classes. The relative abundance is expressed as the fold change of ln(SMFG)—ln(LMFG). If multiplied by 100 the fold change represents the percentage difference of the SMFG compared to the LMFG group. Statistical significance was determined by two-way ANOVA including the studied groups (LMFG and SMFG cows), days in milk and season as fixed effects. *P *values were adjusted for multiple comparisons according to the Benjamini and Hochberg^58^ procedure.

### Differences in polar lipid species

**Figure 3:**
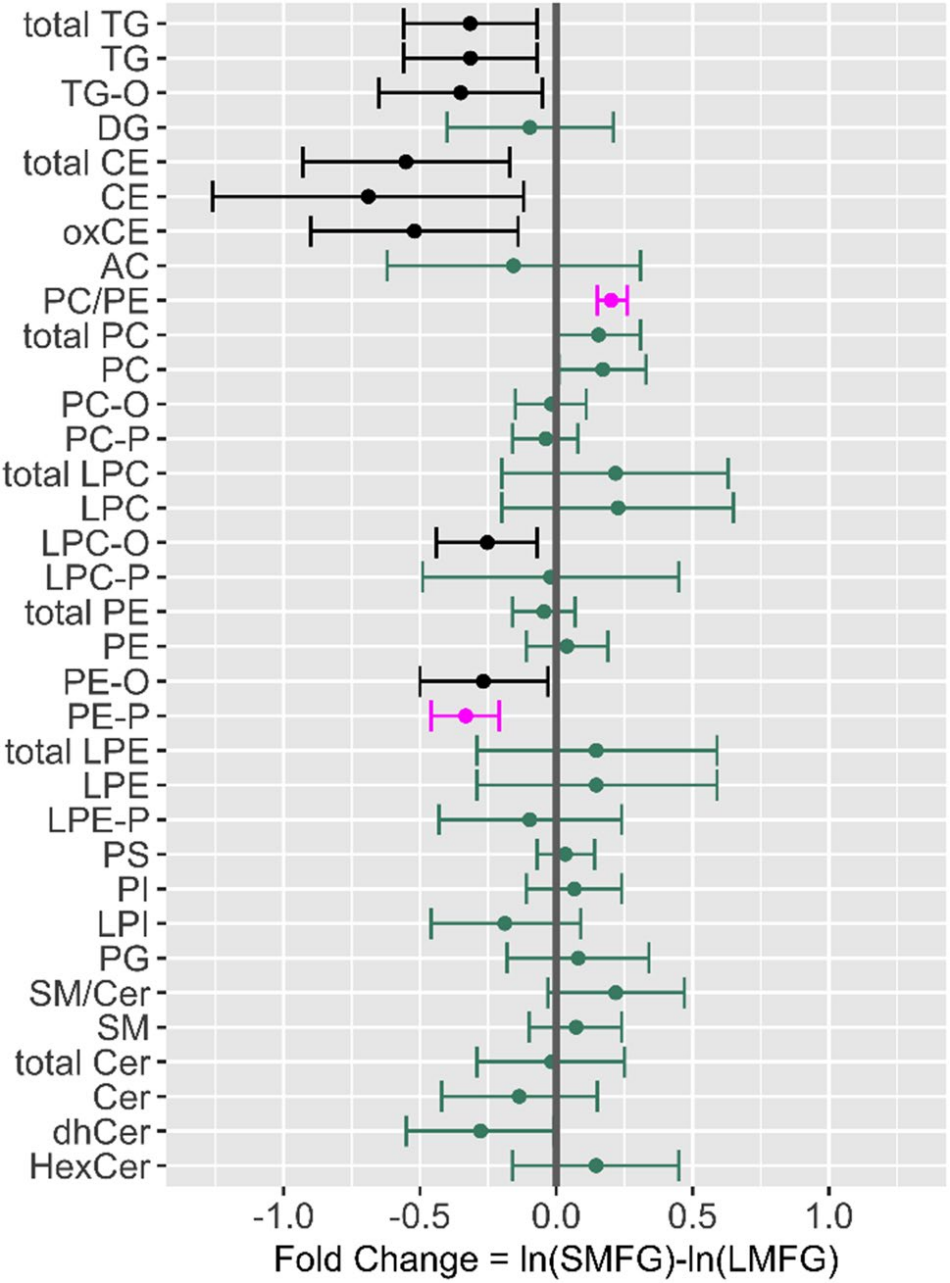
The relative difference in abundance of lipid classes and subclasses in milk from cows with small average milk fat globule size (SMFG) compared to cows with large average milk fat globule size (LMFG). Estimated mean difference and 95% confidence intervals are shown. Lipid classes shown in black (*P* < 0.10) or pink (*P* < 0.05) are different between the groups after adjusting the *P *values according to the Benjamini and Hochberg^58^ procedure.

Comparative analysis of the polar lipid species in raw milk from SMFG and LMFG cows revealed differences in the two major PL classes, PE and PC (Fig. 2B). Milk from SMFG cows contained relatively more of two PC species (PC 36:4 and PC-P 34:0) along with a trend towards higher relative abundance of total PC compared to LMFG cows (16% increase, *P* = 0.123, Fig. 3). The PC/PE ratio was also calculated to minimise the impact of intrinsic differences in PL abundance in small compared to large MFGs, due to a higher PL/TG ratio in smaller MFGs^3^. Indeed, in the SMFG group, PC/PE ratios were higher than in LMFG cows (21%, *P* = 0.001). Milk from LMFG cows, on the other hand, differed from SMFG milk in seven PE lipid species (five species with *P* < 0.05 and two species with *P* < 0.10, Fig. 2B). Some of the ePE species showed increases in relative abundance of over 45% (PE-P 34:3, PE-P 36:1, PE-P 34:1 and PE-O 34:2, all *P* < 0.01). This led to an increase in the plasmanyl-PE and plasmenyl-PE subclasses of 27% and 33% (*P* = 0.104 and *P* < 0.01, respectively). However, these differences did not lead to a change in the total relative abundance of the total PE lipid class between the groups (Fig. 3). These results suggest, for the first time, a possible link between ePE and MFG size.

Some differences were also observed within the Cer and SM classes (Fig. 2C). More specifically, the milk of LMFG cows contained more Cer (d18:1_14:0, *P* < 0.05) and Cer (d18:2_22:0, *P* < 0.10) than the milk of SMFG cows, while the relative abundance of total Cer was similar between the studied groups (Fig. 3). In contrast, SMFG milk contained a higher relative abundance of Hex2Cer (d18:1_22:0, *P* < 0.10) and two SM species (SM 38:1and SM 40:3, *P* < 0.10) compared to LMFG milk (Fig. 2C). Again, this was not related to a difference in total SM abundance (Fig. 3). However, SM/Cer ratios tended to be numerically higher in SMFG milk compared to the LMFG group, although this did not reach statistical significance (Fig. 3).

### Degrees of saturation within the lipid classes

Lipid droplet fusion not only depends on the physical properties of the main PLs in its monolayer but is also influenced by the FA composition of the acyl chains and their impact on fluidity and packing density, with UFAs predicted to increase fluidity and prevent LD fusion^9^. To investigate possible differences in the degree of saturation within the lipid classes between the studied groups, the data was categorised into three groups containing no double bonds, a total of one double bond or a total of two or more double bonds in their acyl chains and the results are shown in Table 3. Numerically, the degree of unsaturation appeared to be higher in some of the polar lipid classes (PC, LPC and PI) along with a numerically reduced degree of saturation within the TG class in the milk from SMFG cows compared to the milk from LMFG cows (Table 3), but these results did not reach statistical significance after adjustment for multiple comparisons. The FA composition of the MFG core (mainly containing neutral lipids), was the subject of our previous study and also showed a higher proportion of SFAs in the core of MFGs form LMFG cows compared to SMFG cows^21^, suggesting that the FA composition of the neutral lipid core could impact MFG size. However, the polar lipids, which are found in the MFGM are more likely to affect LD fusion and in the current study, the degree of saturation within the polar lipid classes did not differ significantly between the studied groups.

## Discussion

Comparative analysis of the lipidomic profiles of milk from the SMFG and LMFG groups showed several important differences, mainly within the PC and PE lipid classes. The milk lipidome from LMFG cows was characterised by a higher relative abundance of plasmanyl- and plasmenyl-ethanolamine lipid subclasses, while the milk from SMFG cows showed higher PC/PE ratios and tended to have a higher relative abundance of total PC species (Fig. 3). The biophysical properties of PC compared to PE result in its superior surfactant properties and increased PC/PE ratios in the monolayer surrounding the intracellular LD are predicted to limit LD fusion^7,8^. Moreover, ePL species and particularly plasmenyl-PE have been shown to reduce the fluidity of biological membranes and have been implicated in membrane fusion events^30,31^. Therefore, the milk lipidome from cows presenting small and large phenotypes provides several differences, which could explain their characteristic MFG phenotype.

A limitation of our study was that the selected cows differed slightly in days in milk and were significantly different in terms of their parities (Table 1). Both these parameters can affect MFG size and could be contributing factors to the observed difference. However, we previously estimated the effect of these parameters on MFG size within our herd^20^. Based on this analysis we estimate that the difference between the groups attributable to the difference in days in milk and parity is in the magnitude of 0.21 µm (when comparing SMFG cows to LMFG cows, a decrease of 0.05 µm can be attributed to 25 additional days in milk in average for SMFG cows; and an additional decrease in MFG size of 0.16 µm can be attributed to the difference in parity, with an average parity of 1.8 for SMFG cows compared to 3.8 in LMFG cows). The difference in MFG size between the selected groups (1.63 µm) is therefore approximately 8 times the estimated effect of the impact of days in milk and parity. However, days in milk and season were added to the statistical model as a fixed effect to control for their impact on MFG size. Nonetheless, due to the lack of studies using LC–MS/MS based lipidomics in milk, at this stage, we cannot determine, to what extend the difference in parity could have contributed to the differences in the lipidomic profiles seen between the studied groups.

The significance of our results lies in the physical properties of ePLs, particularly plasmenyl-PE, and their potential effect on LD fusion. Ether-PLs (ePLs) contain an ether linked fatty alcohol at the *sn*-1 position, where their diacyl equivalents contain an ester linked FA^32^. Among ePLs, there are two subcategories depending on the nature of the ether bond. The so-called plasmanyl-species contain a 1-*O*-alkyl ether bond, while plasmenyl-species have a vinyl-ether bond (*cis* double bond at the Δ1 position adjacent to the ether bond) and are also known as plasmalogens^33^. Plasmalogens are mainly found in the form of PE species and their concentrations are reported to reach up to 18% of the total PLs in membranes and can constitute up to 70% of the total PE lipid class depending on the tissue^33^. However, information on the abundance of plasmalogens in the lactating mammary gland is lacking and can only be estimated based on their abundance in milk PLs, which are derived from the plasma membrane of the mammary epithelial cell. These results suggest an abundance of plasmenyl-PE in cow’s milk of approximately 4.5% of total PLs.

Plasmenyl-PE with its vinyl-ether bond has been shown to increase the speed of fusion in vitro in small unilamellar vesicles^30^. This is attributed to the propensity of plasmenyl-PE to form so called non-bilayer structures at lower temperatures than plasmanyl- and diacyl-PE. Phase transition from the liquid-crystalline to the inverted hexagonal (non-bilayer) state promotes membrane fusion^33^. It is now known that LD fusion contributes to the ultimate MFG size in mammary cells, as shown by in vitro^8^ and in vivo time-lapse imaging^6^. The increased relative abundance of ePE in LMFG cows could also impact packing density and fluidity of the LD monolayer. Ether PLs are more lipophilic, which leads to tighter packing of ether lipids in biological membranes compared to their diacyl counterparts, decreasing membrane fluidity^31^. Membrane fluidity is also predicted to impact LD fusion^34^, with reduced fluidity of the monolayer promoting LD fusion. We therefore suggest that the higher relative abundance of plasmenyl-PE in the milk from LMFG cows, which is predicted to enhance LD fusion, could influence MFG size. This is the first time that a relationship between the ePE subclass and MFG size has been suggested. However, studies focusing on the pathophysiology of ePL deficiencies have revealed various important biological functions. For example, a possible role of plasmalogens in LD homeostasis has been suggested, characterised by smaller LDs in brown adipose tissue of plasmalogen deficient mice^35^.

In terms of the major polar lipid classes, where SMFG cows showed higher PC/PE ratios compared to LMFG cows, the different physical properties of PC compared to PE, as well as some in vitro evidence suggest that PE enhances LD fusion, while PC has a protective effect^7,8,36^. However, several studies on the PL composition of MFGs with different sizes have reported conflicting results^22–24^ or found no difference in the PL composition between small and large MFGs^37^. These studies separated MFGs from bulk raw milk or individual raw milk samples through gravity separation, microfiltration and centrifugation. We used samples from cows naturally different in MFG size distributions, with the aim to study the lipid metabolic differences between the small and large phenotype. Our results support the notion that increased relative PC abundance and more importantly the significantly increased PC/PE ratio are related to smaller MFG size. While the CDP-choline pathway is responsible for the bulk of PC synthesis (about 70%), the phosphatidylethanolamine *N*-methyltransferase (PEMT) pathway (Fig. 1) can make up the remaining proportion^38^ and the PC/PE ratio is regarded as an indicator for the PEMT pathway activity^39^. Notably, during this process PE is converted into PC, resulting in the production of PC at the expense of PE. The PE used during this reaction is predominantly derived from decarboxylation of PS as opposed to PE derived from the CDP-ethanolamine pathway^40^. Moreover, the PC species produced by the conversion of PE into PC contain more long chain and polyunsaturated FAs (PUFAs) compared to the PC species derived through the CDP-choline pathway^41^. Although we found a numerically higher relative abundance of PC species with two or more double bonds (*P* = 0.132, Table 3), this was not statistically significant and we cannot ascertain if the observed trend towards increased relative PC abundance in SMFG cows was due to an increase in unsaturated PC lipid species. Interestingly, it has also been reported that the concentration of PC derived through the PEMT pathway is higher in the LD monolayer compared to other cellular membrane components^40^. Furthermore, the PEMT enzyme has been localised on the surface of LDs or in close proximity to LDs, at sites where they are in close contact with the ER and mitochondria-associated membranes^40^. The contribution of this pathway to the total PC content in mammary tissue is still unknown, but in vitro studies on primary bovine mammary epithelial cells have shown that manipulation of the PEMT pathway affects intracellular LD size^8^. Although the cow’s diet can also affect the relative proportions of the major polar lipid classes^42^, the cows in the current study received the same diet and did not differ in their daily concentrate intake. Therefore, dietary changes are unlikely to be the reason for the observed trend towards higher relative PC abundance and the significantly increased PC/PE ratios in the SMFG group. Whatever the reason for the increased PC content in milk from SMFG cows, the effect of increased PC/PE ratios, and possibly PC species with higher unsaturation, could reduce the propensity of LDs to participate in LD fusion and could impact MFG size.

Our study provides first evidence for a potential role of ePE and confirms previously suggested roles for the proportion of major PLs, particularly the ratio of PC/PE in the size development of MFGs. Our study presents a comparison of the lipidomic profiles of the selected cows and we would like to put forward potential reasons for the observed differences. Firstly, the activity of metabolic pathways that utilise or produce DG could regulate MFG size development in the mammary gland. More specifically, we suggest that the variation in MFG size between the studied groups could be determined by the metabolic activity of the pathways for TG, PL and possibly SL synthesis. The possible relationship between these pathways and MFG size is illustrated in Fig. 1. Since the same enzymes are responsible for the synthesis of ether lipid species and their diacyl equivalents, the final concentration may depend on the relative abundance of their metabolic precursors. Indeed, alkylacylglycerol, the direct precursor for ePL synthesis, is predicted to stimulate ePL synthesis, while DG stimulates the synthesis of PE and PC^43^. Therefore, increased usage of DG for TG synthesis, possibly due to increased DGAT1 activity, could lead to reduced DG availability for PC and PE synthesis. Accordingly, MFG size has been related to the DGAT1 K232A polymorphism, with the KK genotype showing lower PL/TG ratios (larger MFG) compared to the AA genotype^44^. This is attributed to a higher activity of the DGAT1 K variant resulting in increased fat content^45^ as well as a changed FA composition^46^. The results from these studies align with the phenotypical differences seen between the SMFG and LMFG cows in our previous study, where we found less UFAs, but more C16 FAs in the MFG core of LMFG cows^21^. Accordingly, the K genetic variant is related to an increased content of C16:0 as well as SFAs and reduced contents of unsaturated C18 FAs and C14:0^46^. However, in our study the average fat yield and fat content were similar between the studied groups, which is in contrast to the increased fat content which is usually characteristic for the K variant^47^. Under the assumption of increased DGAT1 activity in LMFG cows, this could lead to the reduced availability of DG for PL synthesis because it is primarily used for TG synthesis. Consequently, the limited availability of DG as a precursor for diacyl-PL synthesis could lead to a shift towards ePL synthesis in LMFG cows. Therefore, we suggest that ePL synthesis could represent a possible salvage pathway, when PL synthesis through the conventional pathways is limited, for example when DG is predominantly used for TG synthesis. The fact that plasmenyl-PE is the dominant type of plasmalogen found in most tissues and is the precursor for plasmenyl-PC synthesis explains why mainly ePE species were relatively more abundant in LMFG cows compared to SMFG cows.

Secondly, the lipidomic profile of the milk from the studied cows also revealed differences in some Cer and SM lipid species. Ceramides have been linked to insulin resistance in cows and humans^48,49^. In addition, Cer and particularly the balance between SM and Cer may also be involved in LD biogenesis and this potential relationship is illustrated in Fig. 1. Some evidence suggests that SL, PL and TG synthesis pathways are interconnected and have important effects on LD composition and size^50^. For example, increased SMS1 activity in hepatocytes has been shown to activate PC sensing mechanisms and increase de novo PC synthesis, channelling DGs towards PC synthesis and away from TG synthesis through the DGAT pathway^51^. Although not statistically significant, the ratio of SM/Cer showed a numerical increase in SMFG compared to LMFG cows (22%, *P* = 0.192). Whether or not an increased SM/Cer could potentially have contributed to the increased PC/PE ratios in SMFG cows will need to be elucidated in future studies.

## Conclusion

The results obtained from PL and SL analyses indicated several differences in the lipidomic profiles between the studied groups and we have suggested possible explanations for the observed differences. Firstly, milk from SMFG cows contained more PC relative to PE compared to LMFG cows, a finding that is in accordance with some of the available literature. However, a novel finding of the current study was that the lipidomic profile of milk from the LMFG group was characterised by increased levels of plasmanyl- and plasmenyl-PE. This is the first time that a potential relationship between ether linked PE and MFG size has been suggested, and the underlying mechanisms leading to an increased relative abundance of these lipid species in LMFG cows remains unknown. Moreover, our findings support the hypothesis that in addition to the impact of major lipid classes, especially the relative proportions of the main polar lipid classes, minor lipid classes can also impact MFG size. Therefore, further studies should focus on the precise role of ePL in the mammary gland, their location within the mammary epithelial cell and their absolute abundance, along with the required concentration to exert their suggested biological functions. Overall, the results from the current study suggest a potential relationship between the milk lipidome and the characteristic MFG size of individual cows. Some of these relationships have been suggested for the first time and warrant further investigation. However, it should be noted that the impact of parity on the milk lipidome could not be entirely separated from the observed relationship between the milk lipidome and MFG size. Moreover, we cannot exclude that other metabolic differences between the studied animals, that were not measured in the current study, could have contributed to the individual variation in the milk lipidome of the selected cows.

## Materials and methods

### Cow selection and milk sampling

**Figure 4:**
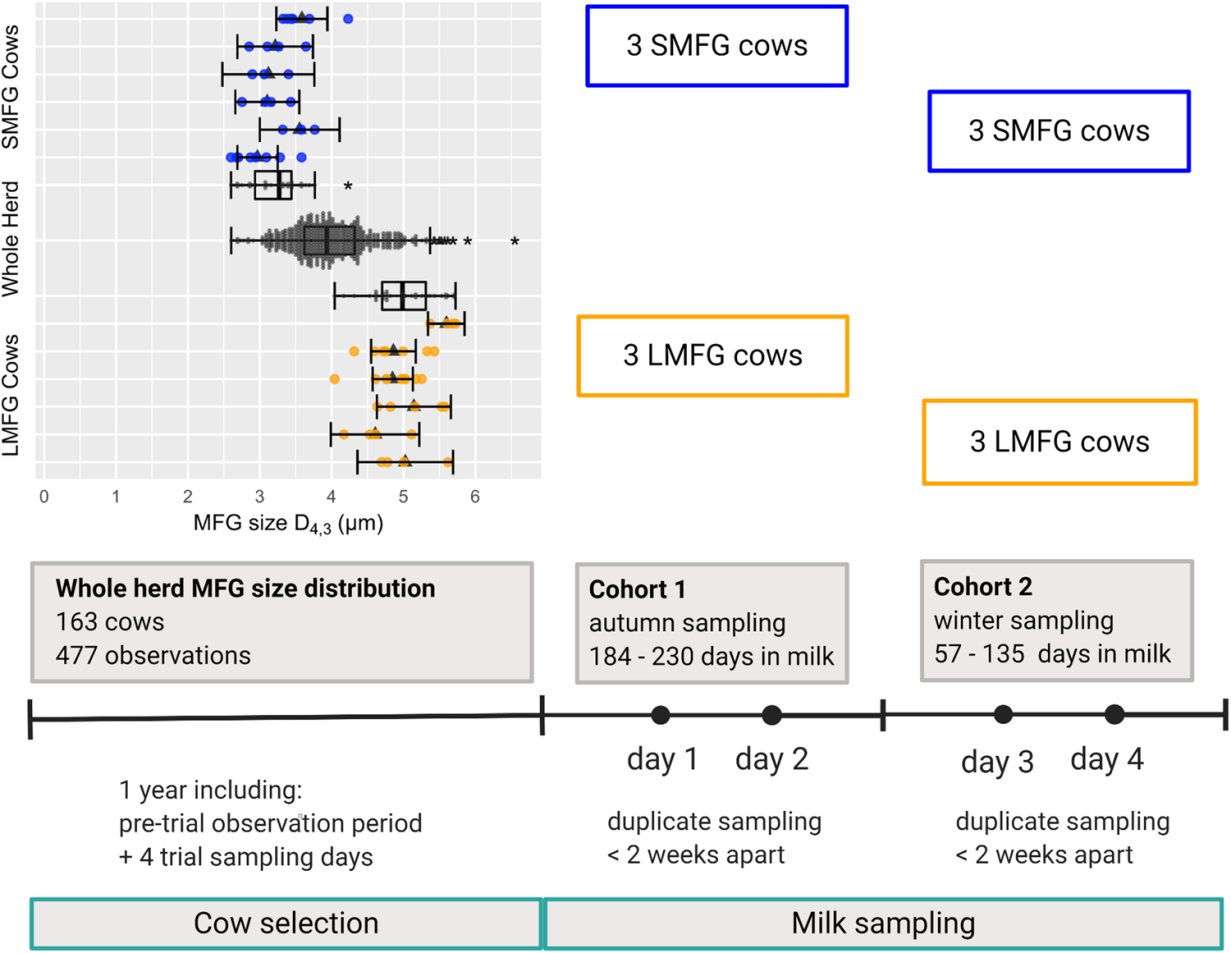
Study design for the current study, including milk sampling and cow selection based on a pre-trial observation period. The top left panel shows the consistency in volume weighted mean diameter D_4,3_ of milk fat globules (MFG) from selected cows compared to the whole herd. Mean symbols (black triangles) and individual values (blue and orange circles for small MFG (SMFG) and large MFG (LMFG) cows, respectively) are shown for the 12 selected cows individually, where error bars represent 95% confidence intervals. Boxplots are shown for the SMFG and LMFG group compared to the data collected for the whole herd including the pre-trial period and the four sampling days of the current study. Asterisks denote outliers 1.5 times the interquartile range above or below the upper or lower quartile, respectively. Each selected cow was measured a minimum of three and a maximum of 9 times. The data presented in the top left panel of this figure is also presented as part of another study^21^ using milk samples from the same cows selected for the current study which were collected at the same time.

A total of 12 Holstein–Friesian cows were selected from the main herd for this study. The cows are part of the herd at the University of Melbourne Dookie Dairy in Victoria, Australia (latitude 36° 25′ 31.3″ S, longitude 145° 42′ 36.6″ E). The herd comprises approximately 150 cows, which are milked by an automatic milking system (Lely Astronaut; Lely, Maassluis, The Netherlands) and pasture fed and kept outside all year. The cows’ diet in the paddock is supplemented with hay and silage if needed and cereal grain-based concentrate is fed during milking. Of the 12 selected cows, six cows consistently produced milk with small average MFG size (< 3.5 µm, expressed as the volume weighted mean diameter D_[4,3]_), forming the SMFG group. The remaining six cows formed the LMFG size group, which produced milk with an average diameter of > 4.5 µm (Fig. 4).

Milk from each selected cow was sampled twice on separate days approximately two weeks apart, resulting in a total of 24 samples from 12 cows. The cows were sampled in two cohorts as illustrated in Fig. 4. The first cohort of six cows, including three per study group, were sampled on two separate days in autumn (March 2016) and the second cohort on two days in winter (June 2016). Milk samples were collected during morning milking (0800 to 1000 h). For the lipidomic analysis, whole raw milk samples (30 mL in 5 ml aliquots) were collected and frozen within 1 min from the end of milking in a liquid nitrogen dry shipper and were subsequently kept at − 80 °C. The University of Melbourne Dookie Dairy is a commercially operating farm and milk samples were taken from the entire milk volume collected from the cows during routine milking.

One of the goals of this study was to select cows that consistently produce milk with average MFG sizes on the small or large end of the spectrum observed within a herd but have otherwise similar milk production traits and are in similar stages of lactation. Therefore, cows were surveyed over one year prior to this study and the selected animals were measured between three to nine times, including the two samples per cow taken for the current study. The MFG size measurements taken of the selected groups compared to the whole herd during the observation period was reported in our previous study^21^.The average MFG size from this pre-trial observation period for the whole herd and the selected groups are shown in Fig. 4. The animals selected for this study were also used for another study looking at the FA profile of the MFG core^21^, where milk samples were collected at the same time as the samples collected for the current work. Therefore, the descriptive data for milk production and animal parameters presented in Table 1 and MFG size measurements for the pre-trial observation period presented in Fig. 4 are identical to the data presented in our other study^21^.

The selected cows differed in MFG size and parity, while milk composition, number of milking sessions per day and concentrate intake were statistically similar between the groups (Table 1). Cows were further selected based on stage of lactation, which resulted in a range of 184–230 days in milk for the first cohort of cows and 57–135 days for the second cohort. Overall, the SMFG cows were, on average, 25 days later in their lactation cycle. Due to the year-round calving pattern in our herd, we decided to perform sampling in two separate seasons in order to obtain two groups which contain animals with the widest possible difference in average MFG size (Fig. 4). Because the goal was to capture the milk lipidomic differences underlying the individual variation in MFG size observed in our herd, season and days in milk were included in the statistical model as fixed effects.

### MFG size measurements and proximate analysis

MFG size measurements and proximate analysis of freshly collected milk samples were performed the day after milk collection following storage in ice water overnight. Fat and protein concentrations were determined using a LactoScope FTIR 20 (Delta Instruments, Melbourne, Australia). MFG size was measured as previously described^13^ with a Mastersizer 2000 particle analyser (Malvern Instruments, Malvern, UK). Prior to particle size analysis milk was mixed 1:1 with 35 mM EDTA, pH 7.0 to dissociate casein micelles and the obscuration rate was kept between 12 to 15%. Refractive indices for milk fat and water were set to 1.46 and 1.33, respectively. Each sample was measured in duplicate and the volume-weighted mean diameter D_4,3_ was used as the measure for the average MFG size.

### Lipidomic analysis and data curation

From 24 milk samples, 20 µL of each sample were used for lipid extraction using a modified Folch extraction protocol^52^. Briefly, 20 µL of milk samples were suspended in 400 µL ice cold chloroform:methanol (2:1, v/v) containing 10 mg/L of each internal standard. The internal standards were PC19:0/19:0, PE-d31, PG17:0/17:0 and TG-d5 19:0/12:0/19:0 (Product # 850,367, 8,609,040, 860,374, and 830,456, from Avanti Polar Lipids, Alabama, USA). Samples were vortexed and then mixed at 950 rpm for 30 min at 27˚C with a Thermomixer C (Eppendorf South Pacific Pty Ltd, Macquarie Park, Australia). Samples were centrifuged at 16,100 × g (Beckman Coulter Microfuge® 22R Refrigerated Microcentrifuge, Beckman Coulter Australia Pty Ltd, Sydney, Australia) for 10 min and the supernatant transferred to fresh LoBind Eppendorf tubes. Samples were completely dried in a vacuum concentrator, with the temperature maintained at 30–35 °C (Christ® RVC 2–33, Martin Christ Gefriertrocknungsanlagen GmbH, Osterode am Harz, Germany). The samples were reconstituted with methanol:water-saturated butanol (100 µL, 1:9, v/v). Pooled biological quality control samples (PBQC) were prepared by pooling aliquots of the extracts from each sample and ran after every five samples.

Extracted lipids were processed and detected by Metabolomics Australia (Bio21 Institute, Melbourne, VIC, Australia) as previously described^27,28^ using an Agilent 1,290 liquid chromatography (LC) system and Triple Quadrupole 6,490 mass spectrometer (MS, Agilent Technologies Australia, Mulgrave, Australia). Briefly, the lipid material (1 μL) was separated using a Zorbax Eclipse Plus C-18 column (100 mm × 2.1 mm × 1.8 µm, Agilent Technologies Australia, 0.4 mL/min) with a mobile phase solvent system of A: water/acetonitrile/isopropanol (50:30:20, v/v/v) and B: water/acetonitrile/isopropanol (1:9:90, v/v/v), both with 10 mM ammonium formate, using a flow rate of 400 µl/min. After equilibration with 10% B, the gradient elution used was 10%-45% B (2.7 min), 53% B (0.10 min), 65% B (0.20 min), 89% B (0.10 min), 92% B (1.9 min), 100% B (1.9 min), then 10% B (1.1 min), total run time 14 min. The column was preconditioned with 10% B for 30 min before a batch analysis. The lipids were detected in positive ionisation mode with dynamic scheduled multiple reaction monitoring. The MS parameters and MRM transitions of each lipid class, subclass and individual lipid species have been previously described^27,28^. Data processing was performed using Agilent’s Mass Hunter Quantitative Analysis (QQQ) software (Agilent Technologies Australia).

### Lipid nomenclature

Lipid species were named according to the LIPID MAPS nomenclature described in Liebisch et al.^53^. Lipid species identified on a group level were named according to their lipid class followed by the total number of carbons and double bonds (e.g. PC 34:1). Where known, the individual FA composition is denoted in brackets separated by an underscore, e.g. PC (18:0_18:1). The underscore signifies that the stereospecific position number (*sn1* or *sn2*) of the FA on the glycerol backbone is unknown. Furthermore, the O- or P- in the PC and PE lipid classes as well as the lysophospholid species designates the ether or vinyl-ether linkages in plasmanyl-and plasmenyl-species, e.g. PE (O-18:1_18:0).

### Statistical analysis

Peak areas of each detected compound were normalised by dividing the respective peak by the aggregate median for all detected compounds (per sample). Ratios of PC/PE and SM/Cer were calculated by calculating the sum of peak areas of the respective lipid class and were not median normalised. The rationale behind calculating PC/PE and SM/Cer ratio was threefold: (1) calculating the ratio of these metabolites can reduce the bias of the normalisation technique (here median normalisation) and resemble the use of an internal standard, which can reduce the impact of biological variability on the data interpretation^54^; (2) calculating the ratio of two metabolites that are the substrate and the product of a biochemical reaction can provide an estimation of the rate of the reaction^54^ and, in our case, can provide an indication of the activity of the reaction between the selected groups; and (3) the PC/PE and SM/Cer ratios have been used as an indicator for membrane integrity^39,55^ and PC/PE is further used as an indicator for PEMT pathway activity in liver lipid metabolism^39^. The data was transformed using the natural logarithm and the mean of duplicate measurements for each cow were used for statistical analysis. Because sampling was performed across two seasons a two-way ANOVA was used for statistical analysis including the studied groups (LMFG and SMFG cows), days in milk and season as fixed effects using the ‘lm’ function combined with the ‘tidy’ function as part of the ‘broom’ package^56^ in R version 3.5.1^57^. The procedure by Benjamini and Hochberg^58^ was used to correct false positives for multiple comparisons and only corrected *P* values are shown. Interactions between the fixed effects were not significant. The estimated difference between the means of the two studied groups based on data transformed using the natural logarithm, represents the fold change of SMFG cows compared to that of the reference group (LMFG cows). If multiplied by 100, the estimated difference between the groups, calculated by the linear model, can be interpreted as a percentage^59^. Although absolute abundance of the compounds was not measured, sums of the median-normalised peak areas of individual lipid species within a lipid class were calculated to estimate the difference in relative abundance of each lipid class. The results of this analysis were transformed using the natural logarithm and significance testing was performed using two-way ANOVA, as described for the individual lipid species, including the correction for multiple testing. Graphs were produced using the ‘ggplot2′ package^60^ in R.

## Supplementary information

Supplementary file1 (PDF 138 kb)

## Data Availability

The datasets generated during the current study are available from the corresponding author on reasonable request.
