# Supplementary material for: Milk fat globule size development in the mammary epithelial cell: a potential role for ether phosphatidylethanolamine
Source: Sci Rep. 2020 Jul 23;10:12299. doi: 10.1038/s41598-020-69036-5 (PMC7378170; doi:10.1038/s41598-020-69036-5)
Supplement: Supplementary file 1 — Supplementary file1 (PDF 138 kb) [file 41598_2020_69036_MOESM1_ESM.pdf]

Supplemental Information

Milk fat globule size development in the mammary epithelial cell: a potential role for ether phosphatidylethanolamine

Leonie Walter,[1,2]* Vinod K. Narayana,[3] Richard Fry,[1] Amy Logan,[2] Dedreia Tull,[3] Brian Leury[1]

1. Faculty of Veterinary and Agricultural Sciences, The University of Melbourne, Parkville, Victoria, Australia 3010
2. CSIRO Agriculture and Food, Werribee, Victoria, Australia 3030
3. Metabolomics Australia, Bio21 Molecular Science and Biotechnology Institute, The University of Melbourne, Parkville, Victoria, Australia 3010

Supplemental Table S1: List of identified lipid species and putative fatty acid composition, where known, for phospholipid classes in milk

Phosphatidylcholine (PC) subclasses			Phosphatidylethanolamine (PE) subclasses		
PC	PC-O	PC-P	PE	PE-O	PE-P[a]
PC 30:0	PC O-32:0	PC P-30:0	PE 32:0	PE O-34:2	PE P-34:1 (16:0_18:1)
PC 31:1	PC O-32:1	PC P-32:0	PE 32:1	PE O-36:3	PE P-34:2 (16:0_18:2)
PC 32:0	PC O-32:2	PC P-32:1	PE 34:1	PE O-36:4	PE P-34:3 (16:0_18:3)
PC 32:1	PC O-34:1	PC P-34:0	PE 34:2	PE O-36:5	PE P-36:3 (16:0_20:3)
PC 32:2	PC O-34:2	PC P-34:1	PE 34:3	PE O-38:4	PE P- 36:4 (16:0_20:4)
PC 34:0	PC O-34:4	PC P-34:2	PE 36:0	PE O-38:5	PE P-36:5 (16:0_20:5)
PC 34:1	PC O-35:4	PC P-34:3	PE 36:1	PE O-38:6	PE P-38:4 (16:0_22:4)
PC 34:2	PC O-36:0	PC P-36:2	PE 36:2	PE O-40:5	PE P- 38:5 (16:0_22:5)
PC 34:3	PC O-36:1	PC P-36:4	PE 36:3	PE O-40:7	PE P-36:1 (18:0_18:1)
PC 34:4	PC O-36:2	PC P-36:5	PE 36:4		PE P-36:2 (18:0_18:2)
PC 34:5	PC O-36:3	PC P-38:4	PE 36:5		PE P-36:3 (18:0_18:3)
PC 36:0	PC O-36:5	PC P-38:5	PE 38:3		PE P-38:3 (18:0_20:3)
PC 36:1	PC O-38:6	PC P-40:6	PE 38:4		PE P-38:4 (18:0_20:4)
PC 36:2	PC O-40:6	PC P-40:7	PE 38:5		PE P-38:5 (18:0_20:5)
PC 36:3	PC O-40:7		PE 38:6		PE P-40:5 (18:0_22:5)
PC 36:4			PE 40:4		PE P-36:2 (18:1_18:1)
PC 36:5			PE 40:6		PE P-36:3 (18:1_18:2)
PC 37:6					PE P-38:4 (18:1_20:3)
PC 38:2					PE P-38:5 (18:1_20:4)
PC 38:3					PE P-38:6 (18:1_20:5)
PC 38:5					PE P-40:5 (18:1_22:4)
PC 38:6					PE P-40:6 (18:1_22:5)
PC 38:7					PE P-38:1 (20:0_18:1)
PC 40:5					
PC 40:6					

PG	PI		PS
PG 34:1	PI 32:0	PI 38:2	PS 36:1
PG 34:2	PI 32:1	PI 38:3	PS 36:2
PG 36:2	PI 34:0	PI 38:4	PS 38:3
	PI 34:1	PI 38:5	PS 38:5
	PI 36:1	PI 38:6	PS 40:5
	PI 36:2	PI 40:4	
	PI 36:3	PI 40:5	
	PI 36:4		

PC-O: plasmanyl-PC, PC-P: plasmenyl-PC (plasmalogen), PE-O: plasmanyl-PE, PE-P: plasmenyl-PE (plasmalogen), PG: phosphatidylglycerol, PI: phosphatidylinositol, PS: phosphatidylserine

[a]Shorthand notation according to Liebisch et al.[1]

Supplemental Table S2: List of identified lipid species for lysophospholipid classes in milk

Lysophosphatidylcholine (LPC) subclasses			Lysophosphatidylethanolamine (LPE) subclasses			LPI
LPC	LPC-O	LPC-P	LPE	LPE-O	LPE-P	
LPC 14:0	LPC O-16:0	LPC P-16:0	LPE 16:0	nd	LPE P-16:0	LPI 18:0
LPC 15:0	LPC O-18:0		LPE 18:0			LPI 18:1
LPC 16:0	LPC O-20:0		LPE 18:1			LPI 18:2
LPC 16:1			LPE 18:2			
LPC 18:2			LPE 20:4			
LPC 18:3						
LPC 18:0						
LPC 18:1						
LPC 20:0						
LPC 20:1						
LPC 20:2						
LPC 20:3						
LPC 20:4						
LPC 20:5						

LPC-O: O-alkyl-linked LPC, LPC-P: O-alk-1-enyl-linked LPC, LPE-O: O-alkyl-linked LPE, LPE-P: O-alk-1-enyl-linked LPE, LPI: lysophosphatidylinositol

Supplemental Table S3: List of identified lipid groups and putative lipid species, where known, for sphingolipid classes in milk

Ceramide subclasses				SM
Cer	dhCer	HexCer	Ganglioside	
Cer (d16:1_16:0)	dhCer 16:0	HexCer (d16:1_18:0)	GM3 (d18:1_16:0)	SM 30:1
Cer (d16:1_22:0)	dhCer 18:0	HexCer (d16:1_24:0)		SM 31:1
Cer (d16:1_24:0)	dhCer 22:0	HexCer (d18:1_22:0)		SM 32:0
Cer (d16:1_24:1)	dhCer 24:0	HexCer (d18:1_24:0)		SM 32:1
Cer (d17:1_22:0)	dhCer 24:1	HexCer (d18:1_24:1)		SM 32:2
Cer (d17:1_24:0)		Hex2Cer (d18:1_22:0)		SM 33:1
Cer (d17:1_24:1)		Hex2Cer (d18:1_24:0)		SM 34:0
Cer (d18:1_14:0)				SM 34:1
Cer (d18:1_16:0)				SM 34:2
Cer (d18:1_18:0)				SM 34:3
Cer (d18:1_20:0)				SM 35:1
Cer (d18:1_22:0)				SM 35:2
Cer (d18:1_24:0)				SM 36:2
Cer (d18:1_24:1)				SM 36:3
Cer (d18:1_26:0)				SM 37:1
Cer (d18:2_16:0)				SM 37:2
Cer (d18:2_22:0)				SM 38:1
Cer (d18:2_24:0)				SM 38:2
Cer (d19:1_16:0)				SM 38:3
Cer (d19:1_18:0)				SM 39:1
Cer (d19:1_22:0)				SM 40:0
Cer (d19:1_24:0)				SM 40:1
Cer (d19:1_24:1)				SM 40:2
Cer (d20:1_24:0)				SM 40:3
				SM 41:0
				SM 41:1
				SM 41:2
				SM 42:1
				SM 42:2
				SM 43:1
				SM 44:1
				SM 44:2
				SM 44:3

Cer: ceramide, dhCer: dihydroceramide, HexCer: hexosylceramide, Hex2Cer: dihexosylceramide, SM: sphingomyelin

Supplemental Table S4: List of identified lipid groups and putative lipid species, where known, for acylcarnitine (AC), cholesteryl ester (CE), diacylglycerol (DG) and triacylglycerol (TG) classes in milk

AC	CE	DG[a]	Triacylglycerol subclasses[b]		
			TG		TG-O[2]
Acylcarnitine 14:0	CE 18:2	DG 30:0 (14:0_16:0)	TG 48:2 (14:0_16:0_18:2)	TG 50:3 (16:1_16:1_18:1)	TG O-50:1
Acylcarnitine 16:0	CE 18:3	DG 32:0 (16:0_16:0)	TG 48:2 (14:0_16:1_18:1)	TG 52:3 (16:1_18:1_18:1)	TG O-52:0
Acylcarnitine 17:0	CE 20:0	DG 32:1 (16:0_16:1)	TG 48:3 (14:0_16:1_18:2)	TG 52:4 (16:1_18:1_18:2)	TG O-52:2
Acylcarnitine 18:0	CE 20:4	DG 34:1 (16:0_18:1)	TG 50:1 (14:0_18:0_18:1)	TG 49:1 (17:0_16:0_16:1)	
	CE 24:0	DG 34:2 (16:1_18:1)	TG 50:4 (14:0_18:2_18:2)	TG 51:0 (17:0_16:0_18:0)	
	CE 24:1	DG 34:2 (16:0_18:2)	TG 48:2 (14:1_16:0_18:1)	TG 49:1 (17:0_18:1_14:0)	
		DG 36:1 (18:0_18:1)	TG 48:2 (14:1_16:1_18:0)	TG 51:1 (17:0_18:1_16:0)	
	oxCE 18:2 +2O NH4	DG 36:2 (18:1_18:1)	TG 50:3 (14:1_18:0_18:2)	TG 51:2 (17:0_18:1_16:1)	
		DG 36:2 (18:0_18:2)	TG 50:3 (14:1_18:1_18:1)	TG 53:2 (17:0_18:1_18:1)	
		DG 36:3 (18:1_18:2)	TG 49:1 (15:0_18:1_16:0)	TG 51:2 (17:0_18:2_16:0)	
		DG 36:4 (18:1_18:3)	TG 51:2 (15:0_18:1_18:1)	TG 54:0 (18:0_18:0_18:0)	
		DG 36:4 (16:0_20:4)	TG 48:0 (16:0_16:0_16:0)	TG 54:1 (18:0_18:0_18:1)	
		DG 38:4 (18:1_20:3)	TG 50:0 (16:0_16:0_18:0)	TG 54:2 (18:0_18:1_18:1)	
		DG 38:4 (18:0_20:4)	TG 50:1 (16:0_16:0_18:1)	TG 54:4 (18:0_18:2_18:2)	
		DG 38:5 (18:1_20:4)	TG 50:2 (16:0_16:0_18:2)	TG 48:1 (18:1_14:0_16:0)	
		DG 38:5 (16:0_22:5)	TG 50:2 (16:0_16:1_18:1)	TG 54:3 (18:1_18:1_18:1)	
		DG 38:6 (18:2_20:4)	TG 52:1 (16:0_18:0_18:1)	TG 54:4 (18:1_18:1_18:2)	
		DG 38:6 (16:0_22:6)	TG 52:2 (16:0_18:1_18:1)	TG 56:6 (18:1_18:1_20:4)	
			TG 52:3 (16:0_18:1_18:2)	TG 58:8 (18:1_18:1_22:6)	
			TG 52:4 (16:0_18:2_18:2)	TG 54:5 (18:1_18:2_18:2)	
			TG 48:3 (16:1_16:1_16:1)	TG 54:6 (18:2_18:2_18:2)	
			TG 50:2 (16:1_16:1_18:0)	TG 56:8 (18:2_18:2_20:4)	

oxCE: oxidised cholesteryl ester

[a]Shorthand notation according to Liebisch et al.[1]

[b]TG-O: can include alkyl-diacylglycerol, dialkyl-acylglycerol or trialkylglycerol.

Supplementary References:

1. Liebisch, G. *et al.* Shorthand notation for lipid structures derived from mass spectrometry. *J. Lipid Res.* **54**, 1523–1530 (2013).
